# Impact of Fungal Endophyte Colonization of Maize (*Zea mays* L.) on Induced Resistance to Thrips- and Aphid-Transmitted Viruses

**DOI:** 10.3390/plants9040416

**Published:** 2020-03-28

**Authors:** Simon Kiarie, Johnson O. Nyasani, Linnet S. Gohole, Nguya K. Maniania, Sevgan Subramanian

**Affiliations:** 1 Department of Seed, Crop and Horticultural Sciences, University of Eldoret, 30100 Eldoret, Kenya; simonkiarie8@gmail.com (S.K.); lgohole@gmail.com (L.S.G.); 2 Plant Health Theme, International Centre of Insect Physiology and Ecology, *icipe*, 00100 Nairobi, Kenya; Johnson.Nyasani@kalro.org (J.O.N.); nmaniania@icipe.org (N.K.M.); 3 Crop Health Unit, Kenya Agricultural and Livestock Research Organization (KALRO), 60100 Embu, Kenya

**Keywords:** maize lethal necrosis, endophytic fungi, *Sugarcane mosaic virus*, *Maize chlorotic mottle virus*, induced resistance

## Abstract

In eastern Africa, Maize lethal necrosis (MLN) is caused by the co-infection of maize plants with *Maize chlorotic mottle virus* (MCMV) (Tombusviridae: *Machlomovirus*) and *Sugarcane mosaic virus* (SCMV) (Potyviridae: *Potyvirus*). With the disease being new to Africa, minimal effective management strategies exist against it. This study examined the potential of 10 fungal isolates to colonize maize plants and induce resistance against MCMV and SCMV. Maize seeds were soaked in fungal inoculum, sown and evaluated for endophytic colonization. Fungus-treated plants were challenge-inoculated with SCMV and/or MCMV to assess the effects of fungal isolates on the viruses in terms of incidence, severity and virus titers over time. Isolates of *Trichoderma harzianum*, *Trichoderma atroviride* and *Hypocrea lixii* colonized different plant sections. All plants singly or dually-inoculated with SCMV and MCMV tested positive for the viruses by reverse transcription-polymerase chain reaction (RT-PCR). Maize plants inoculated by *T. harzianum* and *Metarhizium. anisopliae* resulted in up to 1.4 and 2.7-fold reduced SCMV severity and titer levels, respectively, over the controls but had no significant effect on MCMV. The results show that both *T. harzianum* and *M. anisopliae* are potential candidates for inducing resistance against SCMV and can be used for the integrated management of MLN.

## 1. Introduction

Maize, *Zea mays* L. (Poales: Poaceae), is Kenya’s principal staple food crop and ranks highly in the food security and dietary preferences of many communities [1]. It accounts for 35% of daily calories, and its per capita consumption per year is estimated at 98 kg [2]. In Kenya, maize is produced in almost all agro-ecological zones, either under a monocrop or intercrop system [1], and accounts for approximately 20% of total farm produce from smallholder sector [3].

Insect pests and diseases constitute major biotic constraints leading to poor performance in maize production in Kenya. Recently, a disease termed maize lethal necrosis (MLN) was reported in Kenya [4] and for the first time in Africa. The disease has been reported to spread to other eastern and central African countries such as Tanzania [5], Rwanda [6], Ethiopia [7] and Uganda [8], while MCMV was first reported in Democratic Republic of Congo [9]. MLN causes yield losses ranging from 30% to 100% depending on the crops’ infection stage [10], threatening food security, income and livelihoods in eastern Africa. MLN is a viral disease syndrome that develops when maize or other cereals are co-infected by *Maize chlorotic mottle virus* (MCMV) (Tombusviridae: *Machlomovirus*) and some potyviruses infecting cereals such as *Sugarcane mosaic virus* (SCMV) (Potyviridae: *Potyvirus*) [11]. MCMV is transmitted by insects such as thrips, corn root worms, corn flea beetles, and cereal leaf beetles [12,13,14]. SCMV on the other hand is transmitted by various aphid species [15].

Various management practices such as crop rotation, the use of tolerant hybrids, scouting for disease symptoms and destruction of infected crops, the use of certified seeds, the use of synthetic chemical insecticides to control vectors, weeding to destroy alternate vector hosts, and good agronomic practices to strengthen plants’ resistance against insect pests and diseases have been suggested to curb MLN and its spread [5,16,17]. Recently, the International Maize and Wheat Improvement Centre (CIMMYT) has established at least nine MLN-tolerant hybrids, which have been released or recommended for release in Kenya, Uganda and Tanzania (https://mln.cimmyt.org/mln-resistanttolerant-germplasm/). However, maize hybrids with complete resistance to this newly introduced disease in East Africa are not yet available for use by farmers. Preliminary work indicated that synthetic insecticides have potential to reduce MLN incidence and severity through managing its insect vectors [18]. However, the high costs of these synthetic insecticides, their adverse effects to applicators, environment, non-target organisms and their interference with integrated pest management (IPM) limit their wide use in agriculture [19]. Therefore, there is an urgent need to search for alternative approaches for management of the disease.

Recently, there has been increased focus towards utilization of entomopathogenic fungi (EPF)-based biopesticides for management of insect pests and diseases. EPFs have been reported to occur as endophytes, antagonists of plant pathogens, plant growth promoting agents in association with the rhizosphere [20,21,22]. Fungal endophytes have been reported to protect cereal crops against a plethora of fungal pathogens [23,24,25]. Antagonistic effects of fungal endophytes against viruses have also been reported. Lehtonen et al. [26] observed that the frequency of *Barley yellow dwarf virus* (BYDV) (Luteoviridae: *Luteovirus*) was lowest in endophytically-infected meadow rye grass (*Festuca* (=*Lolium*) *pratensis* Huds.). Jaber and Salem also reported lower virus titer levels of *Zucchini yellow mosaic virus* (ZYMV) (Potyviridae: *Potyvirus*) in squash, *Cucurbita pepo* L., endophyte-inoculated plants with *Beauveria bassiana* (Balsamo Criv.) Vuillemin (1912) isolates as compared to the endophyte-free plants [22]. Muvea et al. [27] reported that endophytically-colonized onion plants induced resistance against onion thrips and thrips-transmitted *Iris yellow spot virus* (IYSV) (Bunyaviridae: *Tospovirus*). However, there is lack of information on the impacts of fungal endophytes against maize lethal necrosis and its inducing viruses. Thus, this study investigates the ability of ten fungal isolates to colonize maize plants and its impact on SCMV and MCMV infection of maize.

## 2. Results

### 2.1. Endophytic Colonization of Maize Plants by Fungal Isolates

**Figure 1:**
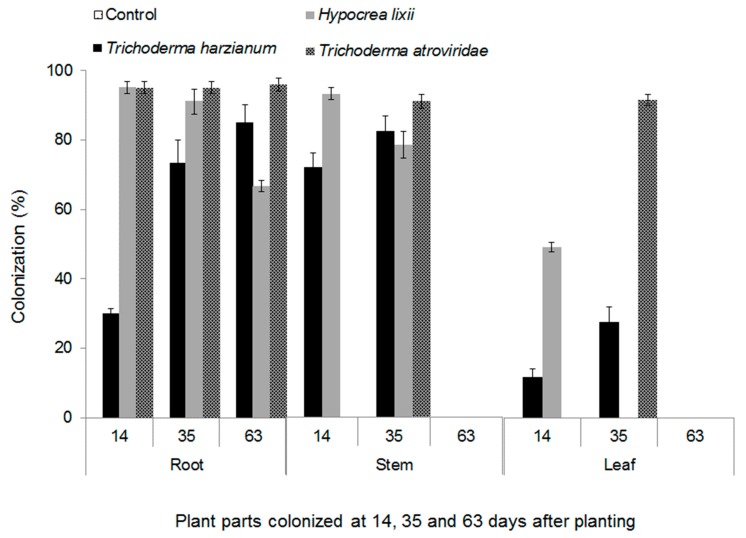
Endophytic colonization (%) of root, stem and leaf sections of maize plants by *Trichoderma harzianum* (F2L4), *Hypocrea lixii* (F3ST1) and *Trichoderma atroviride* (F5S21) at 14, 35 and 63 days after planting. The bars represent means ± SE.

More than 90% of conidia germinated in all the fungal isolates that were used in the experiment. The fungal isolates belonging to *Metarhizium anisopliae* (Metchnikoff) sorokin (that is, ICIPE 20, ICIPE 30 and ICIPE 69) and *Beauveria bassiana* (that is, ICIPE 35, ICIPE 273, ICIPE 279 and G1LU3) did not colonize maize plants; hence, they were not included in data analysis. *Trichoderma harzianum* (Rifai), *Hypocrea.lixii* (Pat) and *Trichoderma atroviride* (P. Karst) colonized all parts (roots, stem, and leaves) of maize plants. There was a significant interaction among fungal isolates and plant parts colonized and different phenological stages (*F*_12,60_ = 15.88, *P* ˂ 0.001). All three fungal endophytes colonized the roots, while some isolates did not colonize the leaves and stems (Figure 1). For example, root colonization was 1.2 and 4.7 times higher than stem and leaf sections, respectively, in *T. harzianum* and 1.5 and 5 times, respectively, in *H. lixii*. In *T. atroviride*, root colonization was three-fold higher compared to both the stems and the leaves. Over time, colonization by *T. harzianum* increased from 14 to 63 days after planting (DAP) for roots and from 14 to 35 DAP for stem and leaves (Figure 1). No colonization of stem and leaves by *T. harzianum* was observed at 63 DAP. On the other hand, colonization by *H. lixii* declined over time from 14 to 63 DAP for the roots, and from 14 to 35 DAP for the stem and leaf. Colonization of the leaves by *H. lixii* was only recorded at 14 DAP and no colonization was observed on the stem and leaf parts at 63 DAP. Root colonization by *T. atroviride* was statistically similar at the different DAP, but stem and leaf colonization were only detected at 35 DAP. At 14 and 63 DAP colonization of the stem and leaf by *T. atroviride* was not detected. 

### 2.2. Effects of Fungus-Treated Maize Plants on Susceptibility to Sugarcane Mosaic Virus Disease

**Figure 2:**
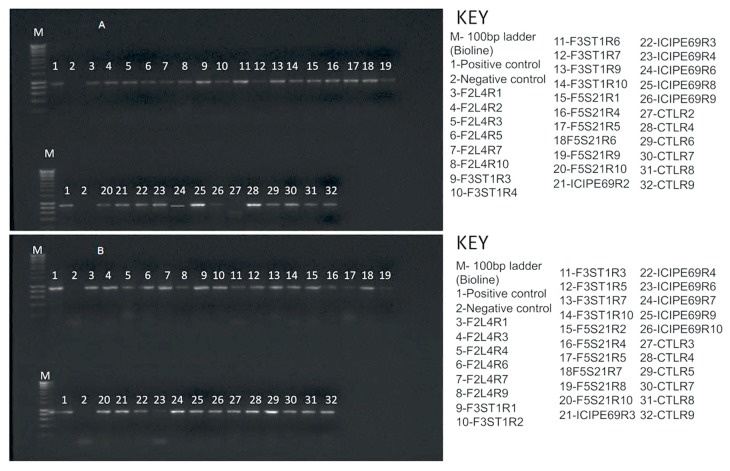
Reverse transcription-polymerase chain reaction (RT-PCR) detection of *Sugarcane mosaic virus* in leaf samples of different fungus-treated plants at 14 days post inoculation. Gels for trials one (**A**) and two (**B**) are presented. F2L4, F3ST1, F5S21 and ICIPE69 represent the fungal endophytes while R1–R10 represent the replicates that were randomly selected for evaluation.

Based on reverse transcription-polymerase chain reaction (RT-PCR) results, almost all representative samples that were artificially inoculated with SCMV tested positive for the virus at 14 days post-inoculation (Figure 2). No virus was detected in the check plants. 

### 2.3. Symptom Development of Sugarcane Mosaic Virus Disease in Fungus-Treated Maize Plants

**Figure 3:**
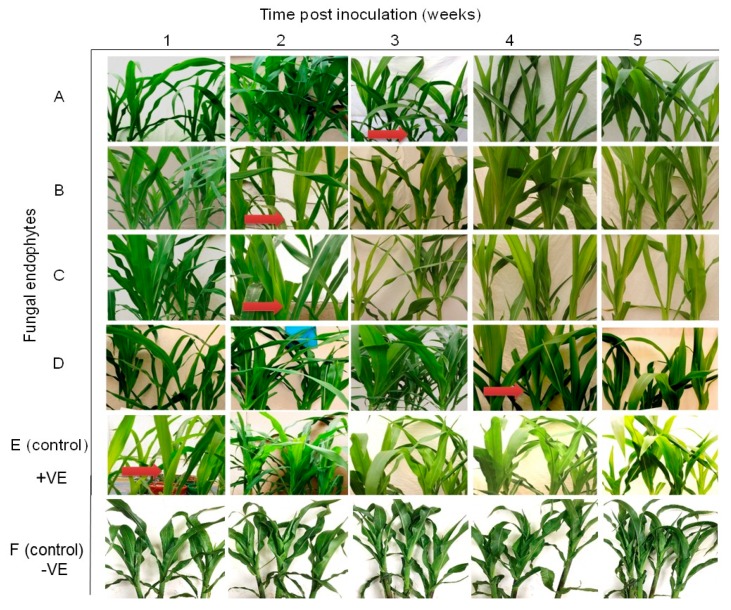
Disease symptom development in maize plants inoculated with fungal isolates, (**A**) (*Metarhizium anisopliae*-ICIPE 69), (**B**) (*Trichoderma atroviride*-F5S21), (**C**) (*Hypocrea lixii*-F3ST1) and (**D**) (*Trichoderma harzianum*-F2L4) following artificial inoculation with *Sugarcane mosaic virus* (SCMV). Panel (**E**) represents the positive control, where untreated maize plants were challenge-inoculated with SCMV, while (**F**) represents the negative control, where plants were neither treated with fungal endophytes nor challenge-inoculated with SCMV. The arrows indicate the onset of symptom expression in various treatments. Challenge inoculation with *Sugarcane mosaic virus* was conducted 21 days after planting.

Differential disease symptom expression was observed in the maize plants treated with the various fungal isolates. Irregular leaf mottling, starting from the base of young leaves, was first observed in the control (fungus-free) plants one-week post-inoculation by SCMV (Figure 3). As time progressed, narrow chlorotic streaks followed by general leaf chlorosis were observed. A delay in symptom expression in fungus-treated plants was observed. Similar symptoms to those observed in control plants at one-week post-inoculation began to manifest in plants inoculated with *H. lixii* and *T. atroviride* at two weeks post inoculation, and in plants inoculated with *M. anisopliae* and *T. harzianum* at three and four-weeks-post inoculation, respectively (Figure 3). Moreover, the symptoms were more pronounced in the positive controls than in fungus-treated plants, whereas no symptoms were observed in the negative control plants throughout the experiment. Symptom development was more obvious in *H. lixii* and *T. atroviride*-colonized plants compared to those colonized by *M. anisopliae* and *T. harzianum* (Figure 3).

### 2.4. Severity of Sugarcane Mosaic Virus Disease

**Figure 4:**
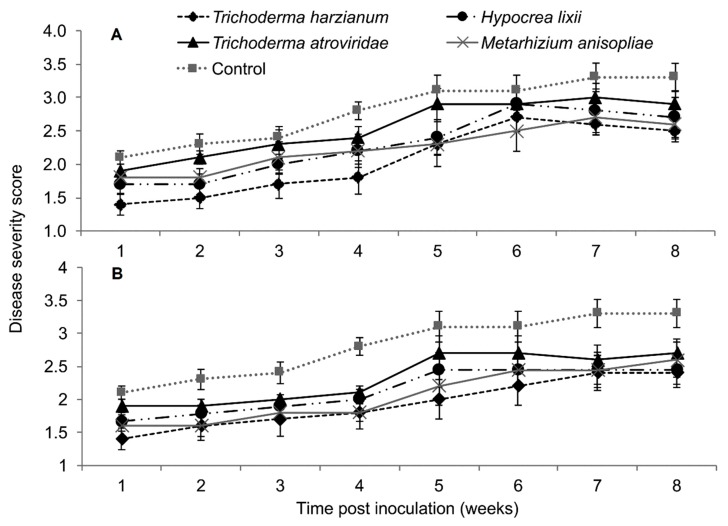
Severity of *Sugarcane mosaic virus* disease as influenced by different treatments over time in trial one (**A**) and two (**B**).

In both trials, the interaction between treatments and time on SCMV severity was not significant as measured by RM-ANOVA (Trial 1: *F*_28,252_ = 0.789, *P* = 0.771; Trial 2; *F*_28,252_ = 0.525, *P* = 0.979). However, the severity of SCMV disease was significantly different in various treatments in both experimental trials (Trial 1: *F*_4,36_ = 2.802, *P* = 0.037; Trial 2: *F*_4,36_ = 4.297, *P* = 0.005). The severity of SCMV was highest in the non-inoculated plants and lowest in *T. harzianum* and *M. anisopliae*-inoculated plants. However, it was lowest in *T. harzianum* as compared to *T. atroviride* throughout the experimental period (Figure 4). Symptom development in plants treated with *T. harzianum* and *M. anisopliae* reduced by 1.4 and 1.2-fold, respectively, over the controls in trial one, while in trial two, both isolates reduced symptom development up to 1.4-fold over the controls. The severity of SCMV was consistently lower in plants inoculated with *T. harzianum* and *M. anisopliae* and highest in the non-inoculated plants, especially in the second experimental trial (Figure 4).

### 2.5. Titer Levels of Sugarcane Mosaic Virus

**Figure 5:**
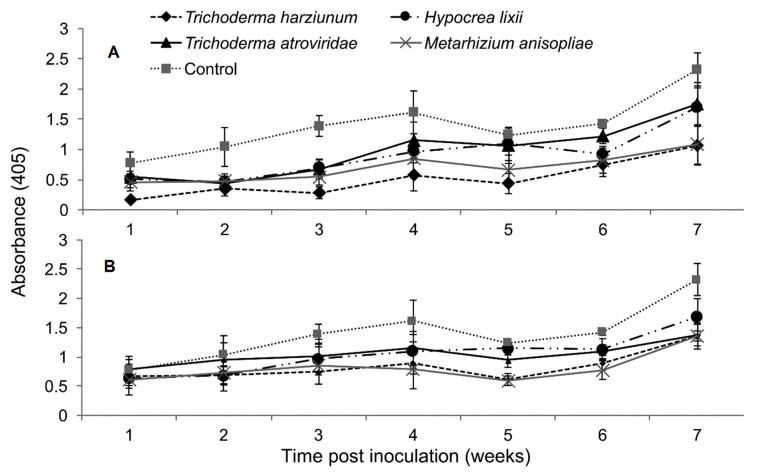
Titer levels of *Sugarcane mosaic virus* estimated over time in maize through ELISA as influenced by different treatments in trials one (**A**) and two (**B**).

The interaction between treatments and time on SCMV titer was not significantly different as measured by RM-ANOVA in both trials (Trial 1: *F*_24,216_ = 0.878, *P* = 0.632; Trial 2: *F*_24,216_ = 1.022, *P* = 0.438). However, the titer levels of SCMV were significantly different in the different treatments of fungus-treated and control plants in both trials (Trial 1: *F*_4,36_ = 4.605, *P* ˂ 0.003; Trial 2: *F*_4,36_ = 2.633, *P* ˂ 0.0464). In trial one, the titer levels of SCMV in plants inoculated with *T. harzianum* and *M. anisopliae* was reduced up to 2.7 and 2.0-fold, respectively, while in the second trial, the two fungal isolates reduced the titer levels of SCMV by 1.7-fold compared to the untreated controls (Figure 5).

### 2.6. Effects of Fungus-Treated Maize Plants on Susceptibility to Maize Chlorotic Mottle Virus Disease

**Figure 6:**
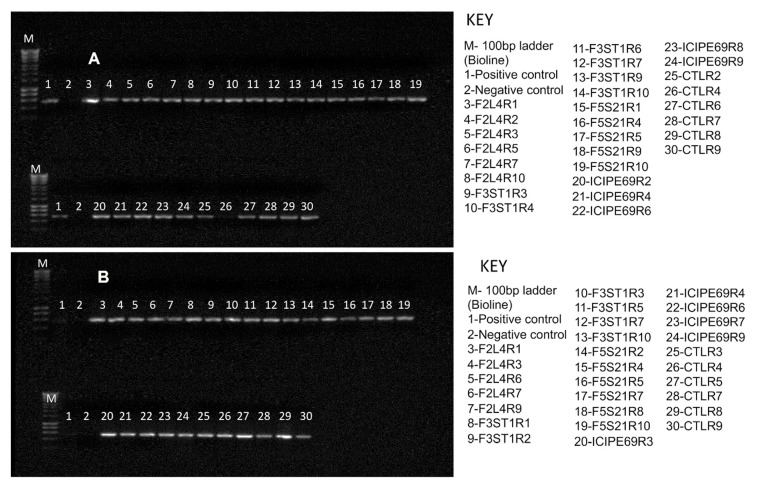
RT-PCR detection of *Maize chlorotic mottle virus* (MCMV) in leaf samples of various treatments at 14 days post inoculation. Gels for trials one (**A**) and two (**B**) are presented. F2L4, F3ST1, F5S21 and ICIPE69 represent the fungal endophytes, while R1–R10 represent the replicates that were randomly selected for evaluation.

Based on RT-PCR results, almost all representative samples that were artificially inoculated with MCMV tested positive for the virus at 14 days post inoculation (Figure 6). No virus was detected in the check plants that were not inoculated with MCMV.

### 2.7. Symptom Development of Maize Chlorotic Mottle Virus Disease in Fungus-Treated Maize Plants

**Figure 7:**
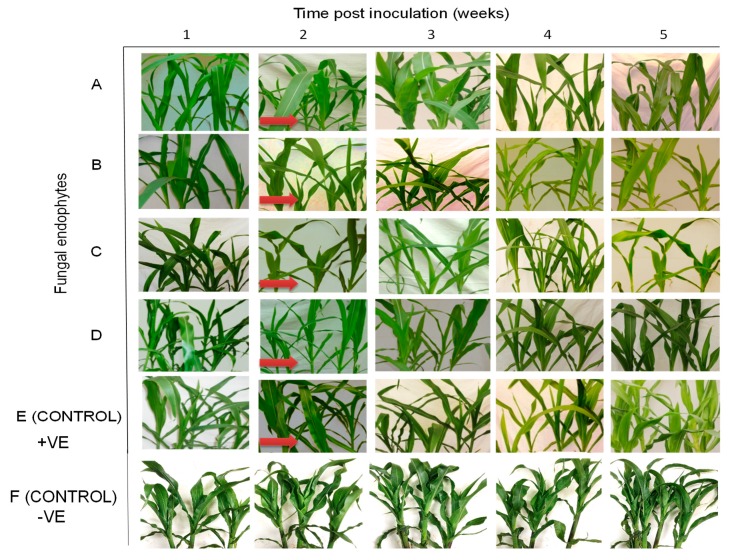
Disease symptom development in maize plants inoculated with fungal isolates, (**A**) (*Metarhizium anisopliae*-ICIPE 69), (**B**) (*Trichoderma atroviride*-F5S21), (**C**) (*Hypocrea lixii*-F3ST1) and (**D**) (*Trichoderma harzianum*-F2L4) following artificial inoculation with *Maize chlorotic mottle virus*. Panel (**E**) represents the positive control, where untreated maize plants were challenge-inoculated with SCMV, while (**F**) represents the negative control, where plants were neither treated with fungal endophytes nor challenge-inoculated with SCMV. The arrows indicate the onset of symptom expression in various treatments. Challenge-inoculation with MCMV was conducted 21 days after planting.

All treatments (including the control plants) were asymptomatic at one-week post challenge-inoculation with MCMV. Fine chlorotic spots begun to appear two weeks post challenge-inoculation with MCMV in the control plants as well as in the plants inoculated with *H. lixii*, *T. atroviride* and *M. anisopliae* (Figure 7). Similar symptoms appeared three weeks post inoculation in plants inoculated with *T. harzianum*. At four weeks post challenge-inoculation, leaf chlorosis was observed in all the treatments. However, the symptoms were slightly more pronounced in the positive controls compared to fungus-treated plants, whereas the negative control plants had no symptoms (Figure 7).

### 2.8. Severity of Maize Chlorotic Mottle Virus Disease

**Figure 8:**
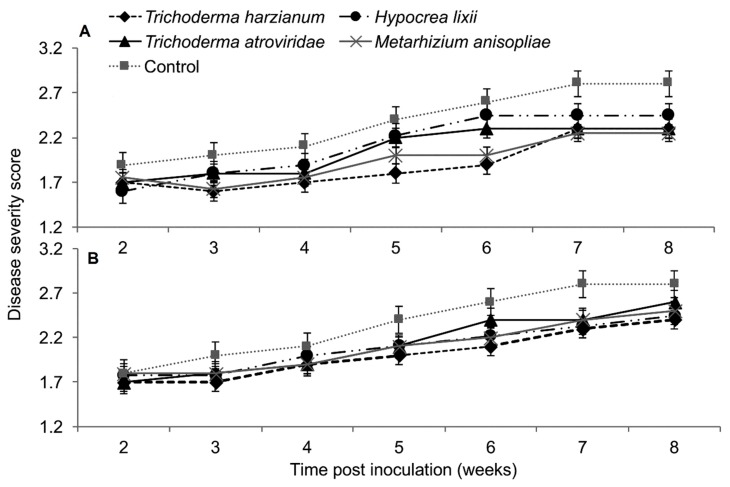
Severity of *Maize chlorotic mottle virus* disease as influenced by different treatments of fungal isolates over time in trials one (**A**) and two (**B**). Challenge inoculation with MCMV was conducted 21 days after planting.

Based on RM-ANOVA, there was no significant interaction between treatments and time on MCMV severity (Trial 1: *F*_28,252_ = 0.463, *P* = 0.987; Trial 2: *F*_28,252_ = 0.463, *P* = 0.987). Additionally, the severity of MCMV was not significantly different in maize fungus-inoculated and control plants in both trials (Trial 1: *F*_4,36_ = 0.988, *P* = 0.424; Trial 2: *F*_4,36_ = 0.988, *P* = 0.424). However, MCMV severity in plants inoculated with *T. harzianum* was consistently lower than in the untreated plants, especially in trial one (Figure 8). 

### 2.9. Titer Levels of Maize Chlorotic Mottle Virus

**Figure 9:**
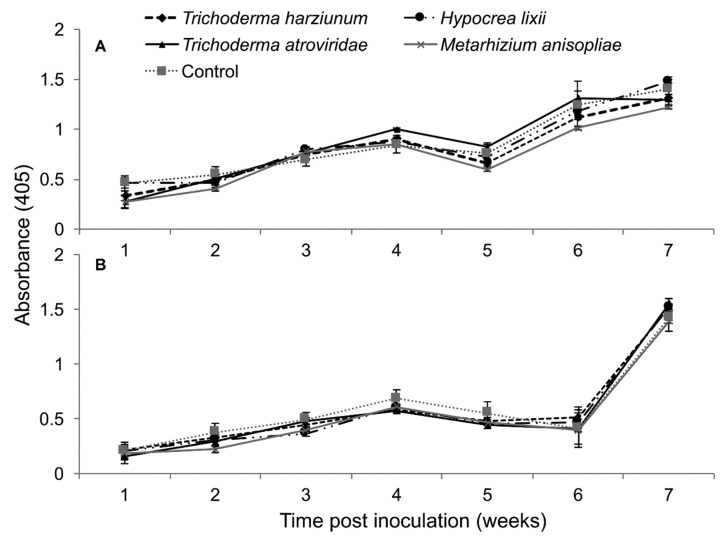
Titer levels of *Maize chlorotic mottle virus* estimated over time through ELISA as influenced by different treatments of fungal isolates in trials one (**A**) and two (**B**).

The interaction between treatments and time on the titer levels of MCMV was not significant by RM-ANOVA (Trial 1: *F*_24,216_ = 1.48, *P* = 0.073; Trial 2: *F*_24,216_ = 0.926, *P* = 0.567). Among the different treatments of fungus-treated and control plants—i.e., *T. harzianum, T. atroviride*, *M. anisopliae* and *H. lixii*—no significant differences in titer levels were observed (Trial 1: *F*_4,36_ = 1.948, *P* = 0.119; Trial 2: *F*_4,36_ = 2.521, *P* = 0.054) (Figure 9). 

### 2.10. Effects of Fungus-Treated Maize Plants on Susceptibility to Maize Lethal Necrosis 

**Figure 10:**
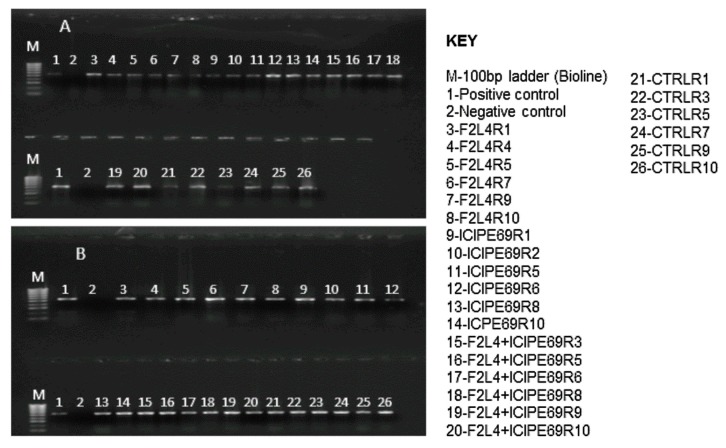
RT-PCR detection of *Sugarcane mosaic virus* (**A**) and *Maize chlorotic mottle virus* (**B**) in leaf samples of various treatments at 14 days post co-inoculation. F2L4 and ICIPE69 represent the fungal isolates, while R1–R10 represent the replicates that were randomly selected for evaluation.

All samples from all the plants that were artificially co-inoculated with SCMV and MCMV tested positive for both viruses at 14 days post-inoculation (Figure 10). No virus was detected in the check plants.

### 2.11. Symptom Development of Maize Lethal Necrosis in Fungus-Treated Maize Plants

**Figure 11:**
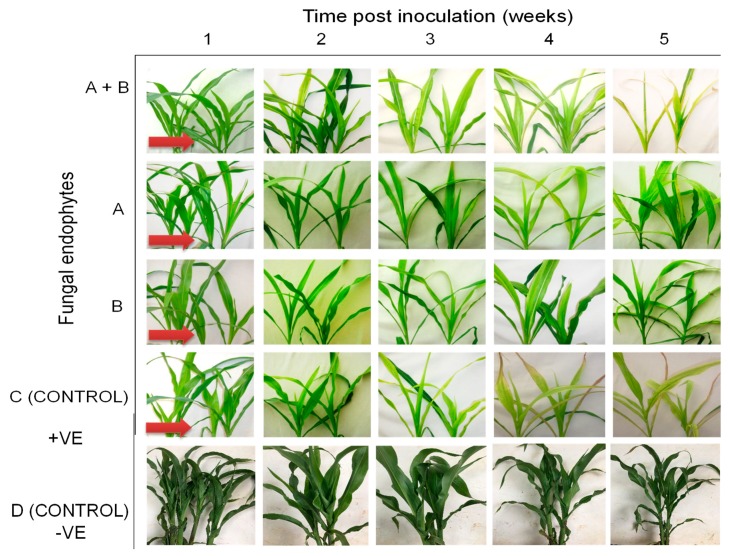
Disease symptom development in maize plants treated with fungal isolates (**A**) (*Trichoderma harzianum*-F2L4), (**B**) (*Metarhizium anisopliae*-ICIPE 69), and A + B (combination of the two isolates-F2L4 and ICPE 69) following dual inoculation with *Maize chlorotic mottle virus* and *Sugarcane mosaic virus*. (**C**) represents the positive control, where untreated maize plants were challenge co-inoculated with MCMV and SCMV, while (**D**) represents the negative control, where plants were neither treated with fungal endophytes nor challenge-inoculated with viruses. The arrows indicate the onset of symptom expression in various treatments. Challenge-inoculation with MCMV was conducted 21 days after planting.

The onset of MLN symptoms was observed one-week post-inoculation in the form of leaf mosaics developing from the bases of young leaves (Figure 11). At three weeks post inoculation, severe chlorosis was observed in the control and in plants colonized by *T. harzianum* and *M. anisopliae*, while chlorotic stripes were observed in plants singly colonized by *T. harzianum* and *M. anisopliae*. Leaf necrosis was observed at four- and five-weeks post-inoculation in the control and in plants colonized by *T. harzianum* and *M. anisopliae*, respectively, whereas severe chlorosis was observed in *T. harzianum* and *M. anisopliae*-colonized plants. No MLN infection symptoms were observed in the negative control plants (Figure 11).

### 2.12. Severity of Maize Lethal Necrosis Disease

**Figure 12:**
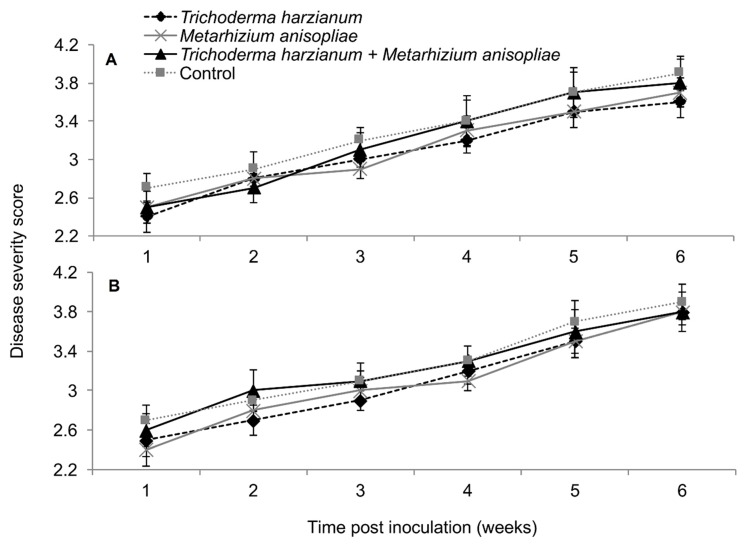
Severity of maize lethal necrosis over time as influenced by different treatments in trials one (**A**) and two (**B**).

In plants dually inoculated with both SCMV and MCMV, early infection symptoms were observed in all the treatments, although leaf necrosis persisted over time in the control plants (Figure 12). The interaction between the treatments and time on disease severity was not significantly different by RM-ANOVA (Trial 1: *F*_15,135_ = 0.445, *P* = 0.963; Trial 2: *F*_15,135_ = 0.719, *P* = 0.763). No significant differences in MLN severity were observed among the various treatments (Trial: *F*_3,27_ = 1.004, *P* = 0.02; Trial 2: *F*_3,27_ = 1.426, *P* = 0.251). 

### 2.13. Titer Levels of Sugarcane Mosaic Virus and Maize Chlorotic Mottle Virus in Dually Infected Maize Plants 

**Figure 13:**
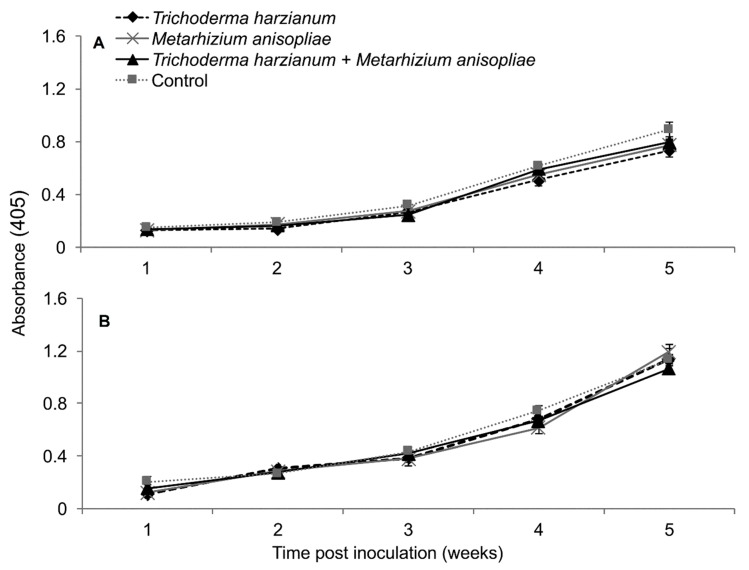
Titer levels of *Sugarcane mosaic virus* (**A**) and *Maize chlorotic mottle virus* (**B**) estimated over time through ELISA influenced by different treatments over time in dually infected plants – First trial.

In the first trial, the interactions between treatments and time on titer levels of SCMV and MCMV were not significant as measured by RM-ANOVA; i.e., (*F*_12,108_ = 1.347, *P* = 0.199) and (*F*_12,108_ = 0.85, *P* = 0.599), respectively. Additionally, no significant differences in titer levels of both SCMV (*F*_3,27_ = 5.883, *P* = 0.002) and MCMV (*F*_3,27_ = 0.971, *P* = 0.417) in different treatments were observed. However, the titer levels of SCMV in controls were distinctly highest from weeks 3–5, whereas those of *T. harzianum* and *M. anisopliae* were lowest, which was not the case with MCMV titers (Figure 13).

**Figure 14:**
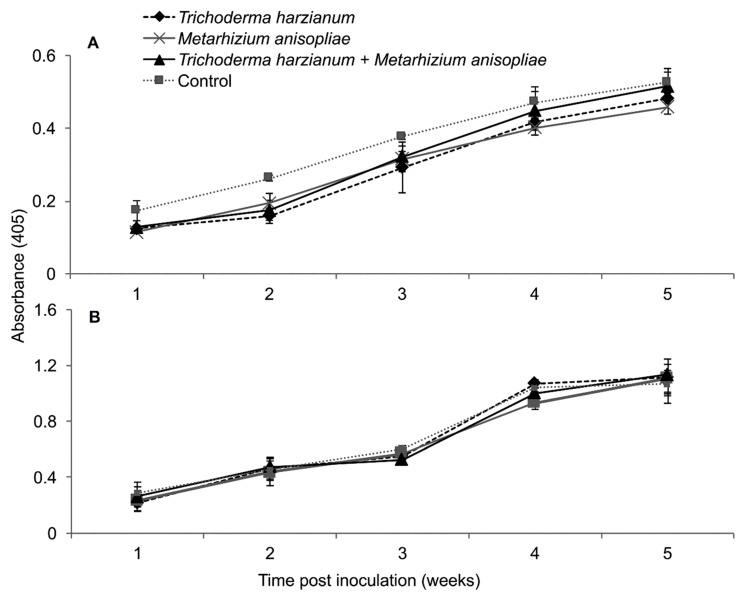
Titer levels of *Sugarcane mosaic virus* (**A**) and *Maize chlorotic mottle virus* (**B**) estimated over time through ELISA influenced by different treatments over time in dually infected plants—Second Trial.

A similar observation that highlighted the above was observed in the second trial, whereby the interactions between treatments and time on titer levels of SCMV and MCMV were not significant as measured by RM-ANOVA; i.e., (*F*_12,108_ = 0.281, *P* = 0.991) and (*F*_12,108_ = 0.51, *P* = 0.906), respectively. No significant differences in titer levels over time of SCMV (*F*_3,27_ = 2.476, *P* = 0.077) and MCMV (*F*_3,27_ = 0.419, *P* = 0.741) were observed in the various treatments of MLN-challenged plants as well (Figure 14). 

## 3. Discussion

Fungal isolates of *T. harzianum, T. atroviride* and *H. lixii* were able to endophytically colonize different parts of the maize plants (roots, stem and leaves) at different phenological stages following seed inoculation. On the other hand, isolates of *B. bassiana* (ICIPE 35, ICIPE 273, ICIPE 279 and G1LU3) and *M. anisopliae* (ICIPE 20, ICIPE 30 and ICIPE 69) did not colonize any parts of maize plants. These findings confirm that endophytic colonization depends on fungal isolates, as reported by other researchers [28,29]. Although fungal isolates belonging to *B. bassiana* and *M. anisopliae* were not found to colonize maize plants in our study, previous reports have highlighted their ability to colonize maize plants [28] or be associated with the plant rhizosphere [30]. The endophytic ability of fungal isolates largely depends on host cultivars [29]. The maize cultivar (H615) used in the present study could be different from that used by Akello [28], which could have contributed to the failure of colonization by the fungal isolates of *B. bassiana* and *M. anisopliae.* In the present study, roots were surface-sterilized prior to screening for endophytic fungi, which could have prevented the observation of root surface colonization if any was present. However, recent evidence based on confocal microscopic techniques with GFP-expressing *Metarhizium* (ARSEF 2575) indicates the potential for root surface colonization by *Metarhizium* sp. [30]. Thus, although some of the EPFs were not found to endophytically colonize the plants, their root surface colonization needs to be evaluated in further detail.

There were differences in the level of colonization of different plant parts—i.e., roots, stem and leaves—by *T. harzianum, T. atroviride* and *H. lixii*, with the highest colonization occurring in the roots followed by the stems, while leaves were the least colonized. Fungi belonging to the genus *Trichoderma* spp. are known opportunistic symbiotic fungi and are widely seen in the soil and known to preferably colonize the plant roots [31]. The marked differences in the colonization levels of different plant parts could be due to tissue specificity as well as adaptation by fungal endophytes to a particular physiological condition present within a given plant tissue [32,33]. This implies that maize roots could have provided more favorable conditions for endophyte survival compared with other plant parts. Similar observations on the colonization of different plant parts in maize [34] as well as other agricultural crops [29,34,35,36] have been previously documented. 

A difference in the colonization pattern of the different fungal isolates over time was observed in the present study. For instance, an overall increase in the colonization of different plant parts was observed in *T. harzianum*-inoculated plants while a decline in colonization was observed in different plant parts inoculated with *H. lixii*. The increase in colonization over time could be due to the rapid growth and perhaps epiphytic spread of *T. harzianum* in maize plants. Crous et al. [37] suggested that the increase in colonization of *Triticum aestivum* (Poaceae Barnhart: *Triticum* L.) over time by fungal endophytes *Alternaria alternata* (Pleosporaceae: *Alternaria*) and *Epicoccum nigrum* (Didymellaceae: *Epicoccum*) was as a result of endophytes’ rapid growth as well as an epiphytic spread in the plant tissues and surfaces, respectively. On the other hand, the decline in the colonization of the plant parts by *H. lixii* over time could be associated with the host response to heterotrophic fungi and competition from other fungi occurring naturally in the soil, which could have competed with the inoculated endophytes for nutrients and space, hence reducing their colonization ability [38]. 

According to RT-PCR results, almost all plants that were inoculated with SCMV acquired the virus; thus, the inoculation exercise was effective, and any variation in disease development was therefore attributed to the effects of fungal isolates. The inoculation of plants with fungal isolates elicited varied levels of protection to maize plants against SCMV in forms of delayed symptom expression, reduced disease severity and reduced virus titer levels over time. To our knowledge, this is the first study in which fungal entomopathogens are reported to confer some protection against *Sugarcane mosaic virus*; a constituent virus inducing MLN disease. The delay in symptom expression, reduced disease severity and reduced titer levels could have resulted from the induced resistance which was triggered by the fungus–plant interaction [39]. Our results corroborate previous studies in which fungal and bacterial agents were used to confer protection against plant pathogens. For instance, a fungal endophyte, *Hypocrea lixii,* was reported to reduce the replication of thrips-borne *Iris yellow spot virus* (IYSP) in onion plants [27]. *Piriformospora indica* (Hymenomycetes: *Basidiomycota*) was reported to induce host defence responses in wheat against *Pseudocercosporella herpotrichoides* (Fron) Deighton through systemic acquired resistance (SAR) [40]. The delay and suppression of symptoms, as well as a reduction in virus titers, were reported in *B. bassiana* isolates against *Zucchini yellow mosaic virus* (ZYMV) in squash plants [22] and *Penicillium simplicium* (GP17-2) (Trichocomaceae: *Penicillium*) against *Cucumber mosaic virus* (CMV) (Bromoviridae: *Cucumovirus*) in *Arabidopsis thaliana* and tobacco [41]. A reduction of SCMV disease symptoms and titer levels could be due to the activation of defense related genes which are triggered by the endophyte–plant interaction. For example, the colonization of *Arabidopsis thaliana* roots by *T. atroviride* induced the overlapped expression of defense-related genes involved in salicylic acid (SA) and Jasmonic acid (JA)/ethylene (ET) pathways which conferred systemic resistance against hemibiotrophic and necrotrophic plant pathogens [42].

The partial protection mediated by the fungal entomopathogens in maize plants could also be due to localization or resistance to virus movement from cell to cell within the plants [43]. Colonization by the fungal endophytes could have interfered with or otherwise restricted the multiplication and local movement of SCMV from cell to cell, hence reducing virus-induced symptoms in those plants. Jaber and Salem [22] suggested that the systemic movement of *Zucchini yellow mosaic virus* (ZYMV) in squash plants was inhibited due to systemic colonization of the plant by strains of *B. bassiana*. Additionally, the fungal metabolites could have been taken up by the plants to the site of viral infection where they directly interacted with the virus hence inducing local resistance [44].

In the present study, induced resistance was also observed with *M. anisopliae,* which was not found to be endophytic in maize plants. Mutune et al. [44] observed that *M. anisopliae* and *B. bassiana* isolates were unable to colonize bean plants; however, *M. anisopliae* (ICIPE 20) proved to be the best fungal isolate in terms of inducing resistance to Bean stem maggot by influencing its lifecycle. Recent evidences indicate the rhizosphere competence [45] and root surface colonization of EPFs such as *M. anisopliae* [30]. The potential for root surface colonization by *M. anisopliae* and its impact on induced resistance cannot be discounted, and this needs to be explored in detail in further studies.

Fungal endophyte-mediated protection against MCMV was not observed in the present study. There were no significant differences in severity and titer levels of MCMV among various treatments. Failure to confer resistance against MCMV could be due to the specificity of the fungal endophytes towards the protection of certain plant viruses. Indeed, some authors have also reported the failure of fungal endophytes to confer resistance against certain plant viruses. For instance, the presence of a grass endophyte, *Acremonium lolii* Latch, Christenson and Samuels, had no effect on BYDV incidence in perennial ryegrass [46]. Although a reduction in BYDV frequencies in tall fescue grass inoculated with an endophyte, *Neotyphodium uncinatum* Gams, Petrini & Schmidt, was reported [26], the elucidated mechanism against the virus was attributed to the poor performance of aphid vectors on endophyte-infected meadow ryegrass rather than the direct influence of the endophyte on the virus. On the other hand, in a recent study, it was reported that volatiles from MCMV-infected maize plants attracted its vector thrips species [14].

No effects of the fungal endophytes were observed on the MLN severity and titer levels of SCMV and MCMV in plants co-inoculated with the two viruses. Furthermore, no evidence of symptom delay was observed in fungus-treated plants relative to the controls. Dual inoculation with SCMV and MCMV usually induces the rapid development of MLN symptoms, which probably could not be suppressed by the fungal endophytes. The development of MLN is due to the increased rate of multiplication of MCMV in the presence of potyviruses such as SCMV [47]. Failure of protection from MLN could therefore be due to this rapid multiplication of MCMV, given the fact that the fungal endophytes did not provide any protection against the virus from our previous results.

## 4. Materials and Methods 

### 4.1. Fungal Isolates

Ten fungal isolates, *Metarhizium anisopliae* (Metchnikoff) Sorokin (3)*, Beauveria bassiana* (Balsamo Criv.) Vuillemin (1912) (4), *Hypocrea lixii* (Pat.) (1), *Trichoderma harzianum* Rifai (1) and *Trichoderma atroviride* P. Karst. (1) were used in this study (Table 1). 

The isolates were obtained from the International Centre of Insect Physiology and Ecology *(icipe)*’s Arthropod entomopathogen germplasm collection. Isolates of *M. anisopliae* and *B. bassiana* were cultured on Sabouraud dextrose Agar (SDA), while the ones of *T. harzianum* and *T. atroviride* were cultured on potato dextrose Agar (PDA) and maintained at 25 °C in complete darkness for 3 weeks as described in Akutse et al. [29]. A spore stock suspension from each isolate was prepared by scraping the surface of the sporulating cultures using a sterile spatula and placing the conidia in 10 mL sterile distilled water containing 0.05% TritonX-100 (Sigma Aldrich, St. Louis, MO, USA). The mixture was vortexed for 5 min to generate homogenous conidial suspensions. Conidial concentration was determined using a Neubauer hemocytometer [48]. The conidial suspension was adjusted to 1 × 10^8^ conidia mL^−1^ through serial dilutions. Conidial viability was assessed by plating 100 µl of the conidia onto 90 mm diameter Petri dishes containing their respective media. Percentage germination of conidia was determined from 100 randomly selected conidia on the surface area covered by a cover slip under a light microscope (40×). Conidia were considered germinated when visible germ tubes longer than half the diameter of the conidia projected from them [48].

### 4.2. Seed Inoculation 

Certified, untreated maize seeds (H615) were surface-sterilized by immersing them in 2% sodium hypochlorite for 2 min and then in 70% ethanol for 2 min. They were rinsed three times in sterile distilled water and spread on sterile paper towels to dry for 10 min under a laminar flow hood. For each batch of fungal inoculum, 30 sterile seeds were soaked in fungal suspensions with 1 × 10^8^ conidia mL^−1^ for 5 h. A cohort of sterilized seeds was also soaked in sterile distilled water for 5 h to serve as controls. Three inoculated seeds were sown per pot (8 cm diameter × 7.5 cm high) containing a sterile mixture of soil and manure at a 5:1 ratio. The soil mixture was sterilized by autoclaving for 2 h at 121 °C and allowed to cool prior to sowing. To neutralize position effects, pots of each treatment was placed in a randomized complete block design (RCBD) in a screen-house with five replicates per treatment.

### 4.3. Evaluation of Colonization by Fungal Isolates 

To determine fungal colonization of different parts of the maize plants at different phenological stages—i.e., leaf development (14 days after planting (DAP)), stem elongation (35 DAP) and flowering (63 DAP) stages [49]—and one plant per pot was carefully sampled, uprooted and washed thoroughly in running tap water. The plants were separated into root, stem and leaf sections. The different plant sections were surface-sterilized as detailed earlier with seeds. The plant sections were aseptically cut under a laminar flow hood into 1 cm × 1 cm each. They were then placed approximately 4 cm apart on 90 mm petri plates with either SDA or PDA media, depending on the fungal isolate, supplemented with a 0.05% solution of antibiotic (*Streptomycin sulfate* salt) and incubated at 25 °C [50]. The last rinse water for seed and plant sections was also plated out on PDA to assess the effectiveness of the surface-sterilization procedure [51]. Ten days after incubation, the presence and growth of the inoculated fungal isolates were evaluated and recorded. Growth of fungal isolates from plant tissues was confirmed by comparing the initially inoculated fungal isolates with the mother plates as described in Muvea et al. [35]. Colonization was recorded by counting the number of pieces of different plant parts showing the growth of the inoculated fungal isolates [35].

### 4.4. Virus Inocula

Virus inocula of *Sugarcane mosaic virus* and *Maize chlorotic mottle virus* were obtained from infected maize plants maintained in separate greenhouses at *icipe*. To prepare virus inoculum, 50 g of each virus-infected leaf was ground with 500 mL of 0.01M phosphate buffer (pH 7.0) using a blender. 20 g of carborundum (silicon carbide, 400–600 mesh) was added to the phosphate buffer prior to inoculation to increase leaf infection by providing minute wounds for entry of the virus particles. 

### 4.5. Mechanical Inoculation with Sugarcane Mosaic Virus and Maize Chlorotic Mottle Virus on Fungus-Treated Maize Plants 

Three of the most effective fungal isolates in colonizing maize plants, *T. harzianum, T. atroviride* and *H. lixii*, and an isolate of *M. anisopliae* (ICIPE 69) were selected for this study. Recent reports indicate that fungal isolates such as *M. anisopliae* have adverse effects on insect pests such as Bean Stem Maggot, *Ophiomyia phaseoli,* though they could not endophytically colonize plants [45]. Hence, an isolate of *M. anisopliae*, ICIPE 69, was also included though it was not an effective colonizer of maize. Maize seeds singly inoculated with the four fungal isolates were sown in pots (20 cm diameter × 18 cm high) filled with sterile soil. Three weeks after germination, the fungus-inoculated maize plants were separately sap-inoculated with SCMV and MCMV in separate screen-houses by the swab method [52]. For each plant, two basal leaves were streaked with virus inoculum four times. Fungus-free plants were also sap inoculated with the viruses to act as a positive control for the experiments. While a negative control, where plants were neither treated with fungal endophytes nor challenge-inoculated with SCMV or MCMV was also maintained in a separate screenhouse. Same negative control plants were used for both SCMV and MCMV infection experiments. Fungus-inoculated plant challenged later with either SCMV or MCMV represented a treatment. Each treatment was replicated ten times, with one plant per pot. The pots were arranged in a RCBD in a screen-house to neutralize position effects. Leaf samples from the third youngest leaves were collected for the detection of SCMV and MCMV by Double Antibody Sandwich-Enzyme Linked Immunosorbent Assay (DAS-ELISA). RT-PCR was also done on leaf samples from six randomly selected plants to detect presence of the inoculated viruses at 14 days post-inoculation.

### 4.6. Mechanical Inoculation with Dual Viruses (SCMV and MCMV) on Fungus-Treated Maize Plants 

Two of the best-performing fungal isolates in the single virus challenge experiment, *T. harzianum* (F2L4) and *M. anisopliae* (ICIPE 69) were selected for use in the dual virus challenge experiment. In this study, synergistic effect of the two isolates (F2L4 and ICIPE69) in reducing MLN incidence and severity was also tested. Different cohorts of maize seeds were separately soaked in fungal inocula of F2L4, ICIPE 69, F2L4 + ICIPE 69 (combined) and in sterile distilled water (positive control). A negative control, where plants were neither treated with fungal endophytes nor challenge inoculated with SCMV and MCMV was also maintained. For combined inoculation, the inocula of the two isolates i.e., F2L4 and ICIPE 69 were prepared as described earlier and mixed in equal volumes. The virus inoculum for SCMV and MCMV were prepared as described earlier, and then mixed at a ratio of 1:1 for dual inoculation. At 21 days after germination, the plants were inoculated with the mixed inoculum using the swab method. Samples were also collected from the third youngest leaves for detection of SCMV and MCMV using DAS-ELISA. RT-PCR was done on leaf samples from six randomly selected plants to detect presence of the inoculated viruses at 14 days post-inoculation. 

### 4.7. Disease Assessment 

Data on disease severity on plants inoculated with single and dual viruses were collected weekly for a period of 8 and 5 weeks, respectively. Disease severity for SCMV and MCMV inoculated plants, was visually rated on a scale of 1 to 5, where 1 = absence of symptoms; 2 = 1–25% of the leaves showing mild mottling and necrosis; 3 = 26–50% of the leaves showing mild mottling, necrosis and yellowing of the leaves; 4 = 51–75% of the leaves showing mottling, necrosis and yellowing of the leaves; 5 = 100% leaves showing severe mottling and necrosis with a dead heart symptom [53]. DAS-ELISA tests were conducted weekly in all plant samples using protocols described in Clark and Adams [54]. Absorbance was quantified using an ELISA reader EPOCH™ microplate spectrophotometer to assess changes in concentration of SCMV and MCMV in different treatments over a period of 7 weeks. Leaf samples for the specific viruses were considered positive when absorption (OD = 405 nm) of the sample wells was twice greater than the mean absorption of the healthy controls.

At 14 days post-inoculation (DPI), six samples from each treatment, which were earlier subjected to ELISA were randomly collected and subjected to Reverse Transcription-Polymerase Chain Reaction [RT-(PCR)] using Master cycler nexus gradient (Eppendorf, Hamburg, Germany) thermal cycler to detect the presence of the inoculated viruses. Total RNAs were purified using RNeasy plant mini kit (QIAGEN, Hilden, Germany) following the manufacturer’s instructions. Complementary Deoxyribonucleic Acid (cDNA) was generated using a High capacity cDNA kit (Applied biosystems, Foster City, CA, USA). The resultant cDNA was used as a template for conventional PCR to amplify a region of the mat peptide and the putative replicase genes for SCMV and MCMV, respectively. PCR was done using a HotStar Taq Master Mix PCR kit (QIAGEN). The primer pairs used for SCMV were 8679F: 5′-GCAATGTCGAAGAAAATGCG and 9595R: 5′GTCTCTCACCAAGAGACTCGCAGC while 2681F: 5′ATGAGAGCAGTTGGGGAATGCG and 3226R: 5′CGAATCTACACACACACACTCCAGC primer pairs were used for MCMV. The conditions for PCR constituted initial denaturation and enzyme activation at 95 °C for 15 min, denaturation at 94 °C for 1 min, annealing at 56 °C for MCMV and 46 °C for SCMV for 1 min and elongation at 72 °C for 1 min. The final extension was done at 72 °C for 10 min after which the samples were held at 4 °C. The PCR products were run on 1% agarose gel electrophoresis.

### 4.8. Data Analysis 

Data on percentage colonization were angular transformed prior to analysis of variance to examine the effect of isolate, plant part and phenological stage of crop on colonization of maize plants. Means separation was done using Student Newman Keuls (SNK) test for cases where factor effects were significant. Data on disease severity and titer levels over time were analyzed using repeated measures analysis of variance (RM-ANOVA). All analyses were performed using R statistical software [55].

## 5. Conclusions

The colonization of maize by the fungal endophytes *T. harzianum, H. lixii* and *T. atroviride* and their association with various parts of maize (root, stem and leaves) demonstrates that these isolates can establish an endophytic relationship with maize, which is not their original host. The abundant occurrence of these endophytes in the roots compared to other plant parts highlights the possibility of the root/rhizosphere colonization of these endophytes. However, differences observed among endophyte strains in terms of stem and leaf colonization need to be further investigated.

Our study indicated that the fungal isolates of *T. harzianum* and *M. anisopliae* conferred some protective role to maize plants against SCMV. This was evidenced by their ability to reduce SCMV disease severity and virus titers over time based on the DAS-ELISA results. On the other hand, the colonization of maize plants by the three endophytes did not confer any protection against MCMV or the dual inoculation of both SCMV and MCMV (MLN). However, further studies based on quantitative PCR (qPCR) techniques will be useful to confirm these findings. Further screening for endophyte strains that could be effective against MCMV could reinforce this resistance. The potential for *M. anisopliae* and *B. bassiana* for the root surface colonization of maize and the mechanisms of induced resistance need to be investigated in detail. Further field experiments on the effectiveness of the two fungal isolates (*T. asperellum* and *M. anisopliae*) in combination or in alternation with synthetic pesticides revealed that the alternated application of synthetic pesticide and fungal products resulted in decreased MLN incidence as compared to the control [56]. However, it is unclear whether this field efficacy is due to any resistance against the virus or due to vector control. Hence, further studies on the virulence of these fungal endophytes against the insect vectors responsible for transmission of MLN viruses, particularly maize thrips and aphids should add more value.

## Figures and Tables

**Table 1:** List of fungal isolates used in this study and their origin.

Fungal Species	Isolate	Source	Origin	Year of Isolation
***Beauveria bassiana***	ICIPE 35	Coffee berry	Kenya	2009
	ICIPE 273	Coleopteran larvae	Kericho, Kenya	2004
	ICIPE 279	soil	Mbita, Kenya	2005
	G1LU3	Monocots	Kenya	2012
***Metarhizium anisopliae***	ICIPE 20	soil	Migori, Kenya	1989
	ICIPE 30	Coleopteran larvae	Kendu Bay, Kenya	1989
	ICIPE 69	Soil	Matete, DRC	1990
***Trichoderma harzianum***	F2L4	Onion	Loitoktok, Kenya	2014
***Trichoderma atroviride***	F5S21	Onion	Loitoktok, Kenya	2014
***Hypocrea lixii***	F3ST1	Napier grass	Kenya	2012
